# P-2113. Development of a Local Multidrug-Resistant Gram-Negative Antibiogram

**DOI:** 10.1093/ofid/ofae631.2269

**Published:** 2025-01-29

**Authors:** Robert Crawford, Courtney Bunner, Jacinta Chin, Alyssa P Gould

**Affiliations:** Novant Health Forsyth Medical Center, Winston-Salem, North Carolina; Novant Health Forsyth Medical Center, Department of Pharmacy, Winston-Salem, NC, Winston-Salem, North Carolina; Novant Health, Charlotte, North Carolina; Novant Health, Charlotte, North Carolina

## Abstract

**Background:**

As gram-negative (GN) resistance continues to rise, it is imperative to understand the best empiric treatment options on a local or regional level. Traditional antibiograms including all isolates may be diluted by sensitive organisms that do not have broad spectrum agents automated for susceptibility testing. The purpose of this review was to develop a health system antibiogram and assess susceptibility rates specific to multidrug-resistant (MDR) GN organisms, including newer broad-spectrum agents that are not routinely reported on traditional antibiograms.

Multidrug-Resistant Gram-Negative Antibiogram 2017-2023

**Figure:**
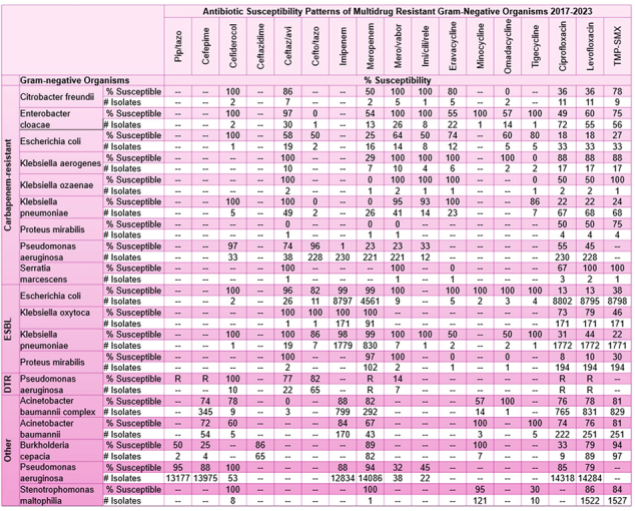

**Methods:**

Data was collected from acute care facilities within a large community hospital system. MDR GN isolates including extended spectrum β-lactamase-producing (ESBL) Enterobacterales, carbapenem-resistant (CR) Enterobacterales, difficult-to-treat (DTR) *Pseudomonas aeruginosa, Acinetobacter baumannii*, *Stenotrophomonas maltophilia,* and *Burkholderia cepacia complex* from January 2017 to December 2023 were included. Susceptibility testing was completed using VITEK 2 system (bioMérieux), E-test or Kirby-Bauer disk diffusion. Interpretation of susceptibility data was based on CLSI M100 guidance.

**Results:**

The MDR GN antibiogram is shown in Table 1. The most common isolates encountered were ESBL *Escherichia coli* (n=8797) and ESBL *Klebsiella pneumoniae* (n=1779). DTR *P. aeruginosa* had the lowest susceptibility across all agents; in comparison to CR *P. aeruginosa* isolates, ceftazidime/avibactam susceptibility was similar however ceftolozane/tazobactam susceptibility was lower. Notably, ceftazidime/avibactam (100%) retained activity for CR *K. pneumoniae* while CR *E. coli* had a lower than expected isolate count and susceptibility. *S. maltophilia* had lower reported susceptibility to trimethoprim/sulfamethoxazole and levofloxacin (84% and 86% respectively) than minocycline (95%) which is performed upon request via manual E-test.

**Conclusion:**

Most MDR GN pathogens within a large community hospital system had reported activity to newer antimicrobial agents. Overall, our antibiogram aligns with treatment recommendations advised by IDSA 2023 Antimicrobial Resistance guidance. Next steps include additional data review for annual susceptibility trends.

**Disclosures:**

All Authors: No reported disclosures

